# In Vivo Acute Toxicity Studies of Novel Anti-Melanoma Compounds Downregulators of hnRNPH1/H2

**DOI:** 10.3390/biom13020349

**Published:** 2023-02-10

**Authors:** Sadeeshkumar Velayutham, Trisha Seal, Samaya Danthurthy, Julia Zaias, Keiran S. M. Smalley, Dmitriy Minond

**Affiliations:** 1 Rumbaugh-Goodwin Institute for Cancer Research, Nova Southeastern University, 3321 College Avenue, CCR r.605, Fort Lauderdale, FL 33314, USA; 2 Department of Pharmaceutical Sciences, College of Pharmacy, Nova Southeastern University, 3321 College Avenue, Fort Lauderdale, FL 33314, USA; 3 Halmos College of Arts and Sciences, Nova Southeastern University, 3301 College Avenue, Fort Lauderdale, FL 33314, USA; 4 Honors College, Nova Southeastern University, 8000 N Ocean Dr., Dania Beach, FL 33004, USA; 5 Division of Comparative Pathology, University of Miami, 1501 NW 10th Ave, Miami, FL 33136, USA; 6 Moffitt Cancer Center, Department of Tumor Biology, 12902 Magnolia Drive, Tampa, FL 33612, USA

**Keywords:** melanoma, drug discovery, spliceosomal inhibition, acute toxicity, blood chemistry, organ histopathology

## Abstract

Despite the recent advances in melanoma therapy, the need for new targets and novel approaches to therapy is urgent. We previously reported melanoma actives that work via binding and downregulating spliceosomal proteins hnRNPH1 and H2. Given the lack of knowledge about the side effects of using spliceosomal binders in humans, an acute toxicity study was conducted to evaluate these compounds in mice. Male and female mice were treated with compounds 2155-14 and 2155-18 at 50 mg/kg/day via subcutaneous injections, and the clinical signs of distress were monitored for 21 days and compared with control mice. Additionally, the effect of the leads on blood chemistry, blood cell counts, and organs was evaluated. No significant changes were observed in the body weight, blood cell count, blood chemistry, or organs of the mice following the compound treatment. The results show that our compounds, 2155-14 and 2155-18, are not toxic for the study period of three weeks.

## 1. Introduction

Melanoma global deaths estimated at approximately 57,000 annually [1,2]. Melanoma mortality rates have decreased between 2013–2019 for the first time since 1975 [3]. This is attributed to the availability of novel therapies: multiple checkpoint inhibitors such as programmed cell death protein 1 (PD-1) as well as cytotoxic T-lymphocyte–associated antigen 4 (CTLA-4) inhibitors and multiple tyrosine kinase inhibitors targeting BRAF–mutated melanoma [4]. However, resistance to both immunotherapy [5,6] and small molecule targeted therapy exists [7], necessitating new targets and approaches to the therapy.

There has been a recent interest in using spliceosomal inhibitors in oncology. Spliceosomal inhibitor Pladienolide B (PlaB) was able to reprogram human committed hematopoietic progenitor cells into multiple progenitors [8], which could be an important tool in reprogramming cancer stem cells. PlaB and its analogs target spliceosomal protein Sf3b [9,10], thereby inducing cell cycle arrest and apoptosis in cervical carcinoma, erythroleukemia and gastric cancer cell lines [11,12,13] A biflavonoid isoginkgetin was shown to inhibit both the major and minor spliceosome by preventing stable recruitment of U4/U5/U6 tri-snRNP, resulting in accumulation of pre-spliceosome A complex [14]. Isoginkgetin was shown to inhibit invasion of fibrosarcoma, breast carcinoma, and melanoma cells in vitro [15] and induced apoptosis and cell cycle arrest in a variety of additional cancer cell lines, with cervical cancer cells being the most sensitive to treatment [16] Phosphorylation of serine/arginine rich splicing factors (SR proteins) is also being investigated as a therapy target. Most studies are focused on SR protein kinases (SRPK1–3) and cdc2-like kinases (CLK1–4) [17], and multiple reports describe Clk inhibitors that display anticancer properties: compound 21b [18], T-025 [19], and CC-671 [20], among many others. To further support clinical development of the CC-671 compound, it inhibited in vivo tumor growth in both a triple-negative breast cancer xenograft model and a patient-derived xenograft model [21]. Two compounds targeting SR protein kinases have shown promise: SRPIN340 and SPHINX [22,23]. SRPIN340 reduced human melanoma tumor growth in vivo but not in vitro, possibly due to the regulation of VEGF expression and angiogenesis reduction [24]. SPHINX inhibited tumor growth in an orthotopic mouse model of prostate cancer [23,25]. Targeting spliceosomes can also induce alternative splicing (AS) [17]. Since AS is dysregulated in many cancers, this can also provide another approach to tackling hard-to-treat cancers, such as melanoma.

We previously reported that the spliceosomal binders we have discovered can induce downregulation of heterogeneous nuclear ribonucleoproteins (hnRNPs) H1, H2, and A2/B1, leading to endoplasmic reticulum (ER) stress, autophagy, and melanoma apoptotic cell death [26]. While the roles of H1/H2 in melanoma have not been extensively studied, there is evidence that members of the H family (H1, H2, and F) contribute to tumor progress and survival in various cancers. In HeLa cells, H1 mRNA targets are enriched in MAPK signaling and ubiquitin mediated proteolysis, which might be the main routes by which H1 promotes tumorigenesis and drug resistance [27]. The H/F complex contributes to drug resistance in glioblastoma [28], while H/F and K contribute to apoptosis resistance in breast tumors via alternative splicing of Mcl-1 [29]. H was shown to facilitate an aberrant splicing of oncogene RON leading to tumor growth [30], and SRSF3 and H1 regulate a splicing hotspot of HER2 in breast tumor cells [31]. hnRNP H drives an oncogenic switch in glioblastoma multiforme (GBM) cells by promoting of aberrant splicing of MADD and RON [32], whereas H1/H2-mediated unsplicing of thymidine phosphorylase results in antitumor drug resistance in leukemia cells [33]. hnRNP H blocks apoptosis by promoting survival in multiple carcinoma cell lines [34], and also promotes colorectal tumor progression by stabilizing the mRNA of Sphingosine-1-Phosphate Lyase [35].

To the best of our knowledge, there were only two clinical trials testing splicesomal inhibitors in which compound E7107 exhibited dose-dependent toxicity and limited efficacy in a Phase I clinical trial [10,36,37]. Given the limited knowledge regarding toxicity of spliceosomal inhibition, we conducted an acute toxicity study using mice to ascertain the safety profile of our lead compounds, 2155-14 and 2155-18.

## 2. Materials and Methods

### 2.1. General Synthesis Procedure for Pyrrolidine-Bis-Diketopiperazine

**Scheme 1:**
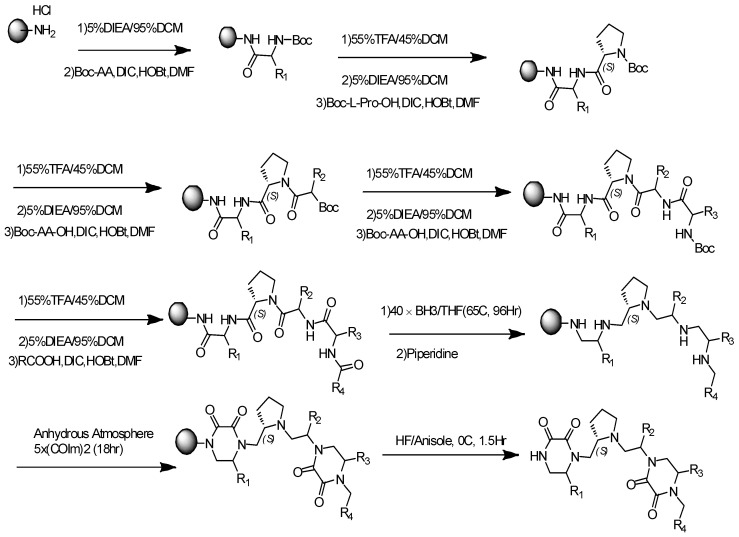
General synthesis procedure for leads of pyrrollidine-bis-diketopiperazine series used in present studies.

The synthesis of Compounds 14 and 18 was previously published by us [15]. All compounds were synthesized via solid-phase methodology (Scheme 1) on 4-methylbenzhydrylamine hydrochloride resin (MBHA) (1.1 mmol/g, 100–200 mesh) using the “tea-bag” approach [38] as previously described [39]. Boc-amino acids were coupled utilizing standard coupling procedures (6 equiv) with hydroxybenzotriazole hydrate (HOBt, 6 equiv), and N,N′-diisopropylcarbodiimide (DIC, 6 equiv) in dimethylformamide (DMF, 0.1 M) for 120 min. Boc protecting groups were removed with 55% trifluoroacetic acid (TFA)/45% dichloromethane (DCM) (1×, 30 min) and subsequently neutralized with 5% diisopropylethylamine (DIEA)/95% DCM (3×, 2 min). Carboxylic acids (10 equiv) were coupled utilizing standard coupling procedures with HOBt (10 equiv) and DIC (10 equiv) in DMF (0.1 M) for 120 min. Completion of all couplings was monitored with a ninhydrin test. Compounds were reduced to polyamines (Scheme 1) using a 40× excess of borane (1.0 M in tetrahydrofuran (THF)) over each amide bond in a glass vessel under nitrogen at 65 °C for 72 h. The solution was then poured off, the reaction was quenched with methanol (MeOH), and the bags were washed with THF (1×, 1 min) and MeOH (4×, 1 min) and allowed to air dry. Once dry, the bags were treated with piperidine overnight at 65 °C in a glass vessel. The solution was poured off, and the bags were washed with DMF (2×, 1 min), DCM (2×, 1 min), MeOH (1×, 1 min), DMF (2×, 1 min), DCM (2×, 1 min), and MeOH (1×, 1 min), and allowed to air dry. Completion of reduction was checked by cleaving a control sample and analyzing using LCMS. As previously reported by our group and others, the reduction of polyamides with borane is free of racemization (NEW REF 1–3). Diketopiperazine cyclization (Scheme 1) was performed under anhydrous conditions (<22% humidity). The dry bags were washed with anhydrous DMF (2×, 1 min), then added to a solution of 1,1′-oxalyldiimidazole (5-fold excess for each cyclization site) in anhydrous DMF (0.1 M) and shaken at room temperature overnight. The solution was poured off and the bags were rinsed with DMF (3×, 1 min) and DCM (3×, 1 min). Completion of cyclization was checked by cleaving a control sample and analyzing by LCMS. The compounds were then cleaved from the resin with hydrofluoric acid (HF) in the presence of anisole in an ice bath at 0 °C for 90 min (Scheme 1) and extracted using 95% acetic acid (AcOH)/5% H_2_O (2×, 5 mL). Final crude products were purified using HPLC as described below. All chirality was generated from the corresponding amino acids. Under the reaction conditions described, no epimerization was observed and, for those compounds with multiple chiral centers, a single diastereomer was obtained.

### 2.2. Compound Purification and Characterization

The final compounds were purified using preparative HPLC with a dual pump Shimadzu LC-20AB system equipped with a Luna C18 preparative column (21.5 × 150 mm, 5 micron) at λ = 214 nm, with a mobile phase of (A) H_2_O (+0.1% formic acid)/(B) acetonitrile (ACN) (+0.1% formic acid), at a flow rate of 13 mL/min; gradients varied by compound based on hydrophobicity. Both 1H NMR and 13C NMR spectra were recorded in DMSO-d6 on a Bruker Ascend 400 MHz spectrometer at 400.14 and 100.62 MHz, respectively, and MALDI-TOF mass spectra were recorded using an Applied Biosystems Voyager DE-PRO Biospectrometry workstation. The purities of the synthesized compounds were confirmed to be greater than 95% by liquid chromatography and mass spectrometry on a Shimadzu LCMS-2010 instrument with ESI Mass Spec and SPD-20A Liquid Chromatograph with a mobile phase of (A) H_2_O (+0.1% formic acid)/(B) ACN (+0.1% formic acid) (5–95% over 6 min with a 4 min rinse) (Supplementary Figures S1 and S2).

### 2.3. Animal Protocol

This study used 5- to 7-week-old male and female Balb/c (Jackson Laboratories) mice. The mice were housed in standard mouse shoe-box cages and maintained in a 12-hr light/12-hr dark cycle, with 50% humidity and 20 ± 3 °C. The mice had free access to a standard pellet diet (Certified PicoLab^®^ Rodent Diet 20, Lab Diet) and water ad libitum. The study was conducted in accordance with the guidelines of the Nova Southeastern University (NSU) Institutional Animal Care and Use Committee (NSU IACUC protocol 2019.12.DM4).

The animals were divided into four groups with each group containing six mice (three male and three female). Group 1: non-treated control mice; Group 2: animals were treated with a vehicle control (10%/90% DMSO/sterile water, USP sterile injectable grade) for 21 days; Group 3: animals were treated with 2155-14 (50 mg/kg body weight) for 21 days; and Group 4: animals were treated with 2155-18 (50 mg/kg body weight) for 21 days three times/week. Compounds (2155-14 and 2155-18) were freshly prepared in 10% DMSO/H_2_O (both USP injectable grade) for each treatment day. Both 2155-14 and 2155-18 were weighed into autoclaved 1.5 mL Eppendorf vials using analytical scales. USP grade DMSO was added to each vial under aseptic conditions and vortexed. USP grade injectable sterile H_2_O was then added to each vial and again vortexed. Next, 1 mL insulin syringes with a 26-gauge needle were filled with 0.2 mL of the compound and delivered to the vivarium in the closed carrier for animal treatment. For the vehicle control group, syringes were filled with 0.2 mL of 10% DMSO/H_2_O (USP injectable grade).

During the experimental period, body weights were measured, and mice were observed for signs of clinical distress every day. More specifically, mice were observed for posture, vocalization, ease of handling, lacrimation, chromodacryorrhea, salivation, coat condition, unsupported rearing, arousal, piloerection, motor movements, diarrhea, tail pinch reaction, and constipation.

All mice were euthanized after 21 days by CO_2_ overdose. Whole blood samples were collected via cardiocentesis with a 25-gauge needle immediately after euthanasia. Blood samples were collected in MiniCollect^®^ Serum and Plasma Tubes containing either K3EDTA or lithium heparin for CBC and blood chemistry, respectively. Blood smears were prepared from whole blood and analyzed for the morphological changes of blood cells under the microscope.

The heart, lungs, stomach, intestines, pancreas, spleen, kidney with adrenal, liver, and brain were collected and placed in 10% neutral buffered formalin for histopathology. The tissues were processed via standard tissue processing to produce H&E slides. All H&E slides were reviewed blind to the treatment group. Tissues were evaluated for the presence of inflammation, degeneration, signs of toxicity, and any other abnormalities. CBC, blood chemistry, and histopathology analyses were performed at the Division of Comparative Pathology, University of Miami.

Statistical significance was set at *p* < 0.05. All data were analyzed using one-way ANOVA to compare means, and significant differences were further analyzed by Tukey’s multiple comparisons using Prism (version 8.0, GraphPad Inc., San Diego, CA, USA).

## 3. Results

**Figure 1:**
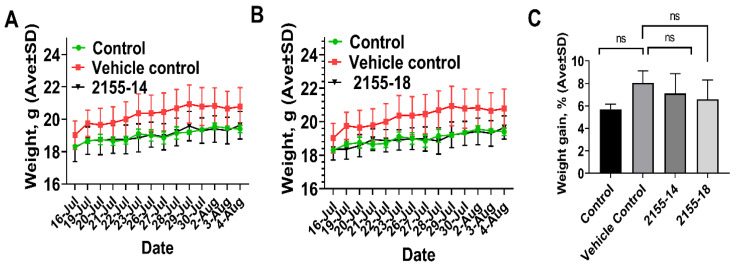
Chemotherapeutic synthetic compounds 2155-14 and 2155-18 administration show a lack of toxic effects on the body weight of Balb/c mice. (**A**) Mice treated with 50 mg/kg/day of 2155-14 show similar to untreated control and vehicle control body weights throughout the study. (**B**) Mice treated with 50 mg/kg/day of 2155-18 show similar to untreated control and vehicle control body weights throughout the study. (**C**) Body weights of mice measured on the final day of the study show overall lack of body weight gain or loss. Mice were injected subcutaneously with 50 mg/kg BW of 2155-14 and 2155-18 3×/week for 3 weeks. No significant changes in body weight were observed in the treated group as compared to non-treated control groups. ns—no significance. Data shown are Mean ± SD (n = 6) analyzed using a one-way ANOVA followed by Dunnett’s test compared with Vehicle Control.

As evidenced by Figure 1, there was no weight loss detected in the treatment groups as compared to the vehicle control group, suggesting the lack of overall gross toxicity. All mice exhibited normal behavior during the study.

Complete blood counts (Table 1) revealed no differences between the treatment groups, suggesting the lack of gross effects on circulating blood cells. In some cases, the counts of mice treated with 2155-14 and 2155-18 were somewhat lower than the counts of the vehicle control group (e.g., eosinophils, basophils); however, they remained within the reference value ranges for the respective parameters. For Hgb, the value for the 2155-18 treated group was below the lower reference limit; however, the difference was not statistically significant (*p*-value < 0.05).

Similarly, examination of blood smears from mice did not reveal any abnormalities in the sizes or shapes of blood cells. Serum blood chemistry analyses were not significantly different between the treatment group as compared to non-treated control group, suggesting no signs of toxicity (Table 2).

**Figure 2:**
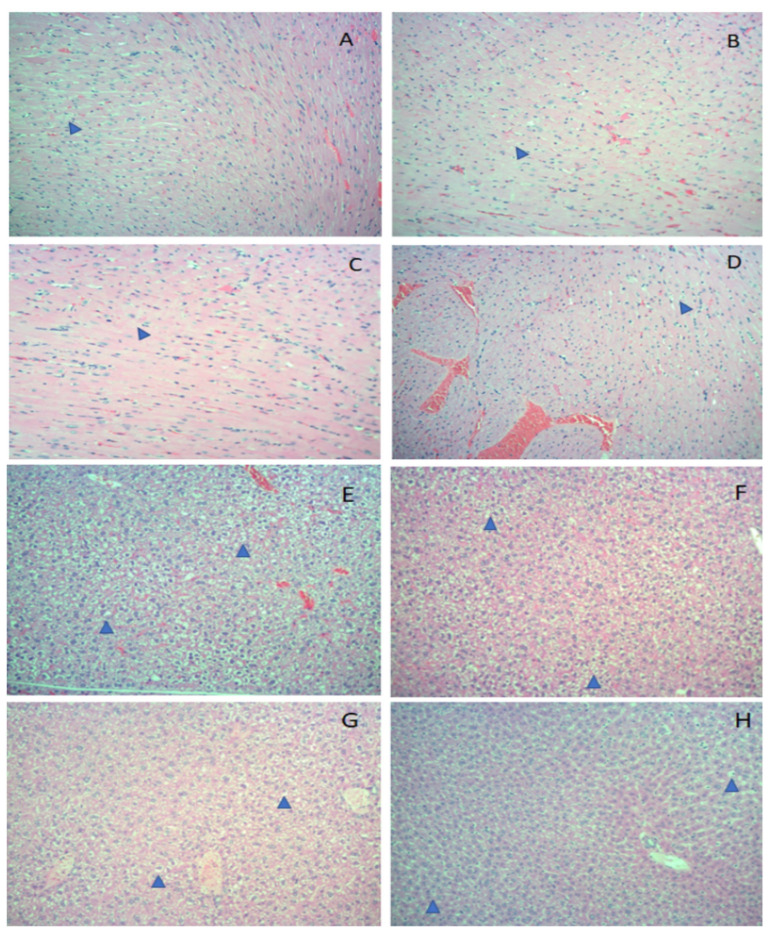
Histopathological examination of hearts and livers of mice treated with 2155-14 and 2155-18 shows no adverse effects. (**A**) Mouse heart untreated control; (**B**) vehicle control; (**C**) 2155-14; (**D**) 2155-18. Blue arrowheads indicate foci of fibrillar disarray myocardium, cardiomyocyte degeneration, diffuse, and moderate. (**E**) Mouse liver untreated control; (**F**) vehicle control; (**G**) 2155-14; (**H**) 2155-18. Blue arrowheads indicate examples of glycogen deposition within hepatocytes, diffuse, and moderate. To take these images, 100× magnification was used.

Overall, there were no significant histopathologic findings in the tissues examined (Figure 2). The hearts of all animals contained fibrillar disarray, nuclear rowing, nuclear pleomorphy, and heterogeneity of fibers, consistent with cardiomyocyte degeneration (Figure 2A–D). Findings of cardiac degeneration are not uncommon in laboratory mice.

The liver in all animals contained mild to marked vacuolization (Figure 2E–H) consistent with glycogen deposition (confirmed by PAS staining).

Congestion and/or alveolar hemorrhage (of varying degrees) in the lungs were observed in most of the animals, which is consistent with agonal changes. The other tissues were not remarkable. Overall, results of CBC, blood chemistry, and histopathology suggest the absence of gross toxicity as a result of sc administration of 2155-14 and 2155-18.

## 4. Discussion

The results of this study demonstrate the lack of acute toxicity after sc administration of two novel anti-melanoma compounds, 2155-14 and 2155-18. We previously reported a lack of general cytotoxicity in 2155-14 and 2155-18 against a wide variety of cell lines and primary cells from different tissues (liver, lung, breast, brain, skin, ovary, primary melanocytes, and primary keratinocytes) [26,40]. Both 2155-14 and 2155-18 exhibited in vitro activity in inhibiting the viability of melanoma cells in the 0.5–10 µM range (IC50 ≈ 0.5–10 µM), while showing no toxicity against various cell types up to 100 µM (TC_50_ > 100 µM), suggesting an in vitro therapeutic index (TI) of ~10. Therefore, it is not entirely surprising that we observed no gross toxicity in BalbC mice in the short-term study even at the relatively high dose (50 mg/kg/day).

2155-14 and 2155-18 belong to the pyrrolidine diketopiperazine chemotype that was extensively screened under the auspices of Molecular Libraries Probe Center Network against a wide variety of targets [41,42] in approximately 200 bioassays (unpublished). Their activity was concentrated only on four targets (one non-human) with sub-micromolar activity, which suggests that these compounds are non-promiscuous. These biological profiling data align well with the lack of acute toxicity in mice.

We previously demonstrated the mechanism of action (MOA) of 2155-14 to be based on binding of the spliceosomal proteins, which are believed to be highly conserved in all tissues due to their central role in controlling pre-mRNA maturation (splicing) [26]. However, the lack of broad-spectrum in vitro cytotoxicity and acute in vivo toxicity suggests the existence of significant differences in either the structure or function of the spliceosomal proteins in melanoma cells that are targeted by 2155-14 and 2155-18. Since 2155-14 and 2155-18 do not have an effect on the viability of primary melanocytes, this further implies that the differences in spliceosomal proteins in melanoma cells are either a cause or an effect of melanomagenesis. Further studies are needed to determine the connection between hnRNP H1/H2 and tumor progression in melanoma.

As stated in the Introduction, new targets and approaches to the melanoma management are needed. Our research is focused on the preclinical characterization of binders of spliceosomal proteins hnRNP H1/H2 as a potential new approach to melanoma management. Our results demonstrate that targeting these proteins does not result in overt toxicity; therefore, this approach should be further evaluated for efficacy as a mono and adjuvant therapy in various animal models and, hopefully, human clinical trials. If successful, targeting spliceosomal proteins hnRNP H1/H2 and other members of the hnRNP protein family could open new venues for cancer research and drug discovery.

## 5. Conclusions

The results presented herein suggest a lack of in vivo toxicity of our lead melanoma actives in a short-term toxicity study in mice. This, in turn, paves the way for future in vivo studies of our leads in various models of melanoma.

## Figures and Tables

**Table 1:** Results of CBC analysis of Balb/C mice treated with 50 mg/kg bw 2155-14 and 2155-18.

Parameter	Control	Vehicle	2155-14	2155-18	Reference Range	Units
WBC	11.4 ± 0.42	6.8 ± 2.5	5.1 ± 1.6	4.2 ± 2.7	3.48–14.03	K/µL
RBC	9.0 ± 1.1	9.6 ± 1.9	10.6 ± 2.1	7.9 ± 1.4	6.93–12.24	M/µL
Hgb	15.5 ± 0.5	15 ± 1.7	14.1 ± 1.6	* 11.9 ± 1.6	12.6–20.5	g/dL
HCT	53.8 ± 4.6	56.8 ± 12.2	61.1±	46.9 ± 6.4	42.1–68.3	%
MCV	59.5 ± 0.7	59 ± 1.4	61 ± 2.2	59.3 ± 2.1	50.7–64.4	fL
MCH	17 ± 1.4	15.6 ± 2.3	14 ± 2.3	14.7 ± 0.57	13.2–17.6	pg
MCHC	28.5 ± 2.1	27 ± 4.2	23 ± 5.1	25.3 ± 0.58	23.3–32.7	g/dL
Segmented Neutrophils	18.5 ± 3.5	23.3 ± 7.2	18.8 ± 8.0	24.7 ± 10.9	9.86–39.11	%
Banded Neutrophils	0	0	0	0	0–1	%
Lymphocytes	73.5 ± 6.4	67.3 ± 8.4	78.3 ± 7.5	70.7 ± 11.3	48.81–83.19	%
Monocytes	4.5 ± 0.7	5.83 ± 2.22	4.25 ± 4.27	6.7 ± 2.3	3.29–12.48	%
Eosinohils	3.0 ± 1.4	2.7 ± 1.2	0.5 ± 1.2	2 ± 1	0.11–4.91	%
Basophils	0.5 ± 0.7	1 ± 0	0	0	0–1.84	%
Platelets	686 ± 279	535 ± 88	542 ± 95	741 ± 305	420–1698	K/µL

Results are averages of 6 replicates ± SD analyzed using a one-way ANOVA followed by Dunnett’s test compared with Vehicle Control. Notes-*—no significance. Reference values are provided by Charles River based on the analysis of their North American Balb/C mice colonies. WBC—white blood cells, RBC—red blood cells, Hgb—hemoglobin, HCT—hematocrit, MCV—mean corpuscular volume, MCH—mean corpuscular hemoglobin, MCHC—mean corpuscular hemoglobin concentration.

**Table 2:** Results of blood chemistry analysis of Balb/C mice treated with 50 mg/kg bw 2155-14 and 2155-18.

Parameter	Control	Vehicle	2155-14	2155-18	Reference Range	Units
Total protein	8.4 ± 3.5	8.1 ± 0.6	7.1 ± 0.6	6.7 ± 0.48	4.6–8.9	g/dL
Creatinine	0.5 ± 0	0.5 ± 0	* 0.44 ± 0.19	* 0.46 ± 0.11	0.2–0.4	mg/dL
BUN	18.5 ± 2.1	17 ± 1.1	18.3 ± 2.5	16.7 ± 1.6	7.0–26	mg/dL
Glucose	170 ± 39.7	199 ± 49.7	181 ± 25.7	212 ± 50.5	129–329	mg/dL
Calcium	10.5 ± 0.25	9.4 ± 0.2	9.1 ± 0.85	8.7 ± 0.75	9.4–12.5	mg/dL
Phosphorus	11.5 ± 0.2	13.2 ± 0.8	13.6 ± 0.4	* 15.3 ± 0.25	8.2–14.7	mg/dL

Results are averages of 6 replicates ± SD analyzed using a one-way ANOVA followed by Dunnett’s test compared with Vehicle Control..Reference values are provided by Charles River based on the analysis of their North American Balb/C mice colonies. Notes-*—no significance. BUN—Blood urea nitrogen.

## Data Availability

Not applicable.

## Supplementary Materials

The following supporting information can be downloaded at: https://www.mdpi.com/article/10.3390/biom13020349/s1, Results of NMR characterization of lead compounds; Figure S1: NMR characterization of compound 14. Figure S2: NMR characterization of compound 18.
