# Supplementary material for: In Vivo Acute Toxicity Studies of Novel Anti-Melanoma Compounds Downregulators of hnRNPH1/H2
Source: Biomolecules. 2023 Feb 10;13(2):349. doi: 10.3390/biom13020349 (PMC9953269; doi:10.3390/biom13020349)
Supplement: Supplementary file 1 [file biomolecules-13-00349-s001.zip › biomolecules-2029829-supplementary.pdf]

biomolecules

MDPI

Brief Report

In Vivo Acute Toxicity Studies of Novel Anti-Melanoma Compounds Downregulators of hnRNPH1/H2

Sadeeshkumar Velayutham [1,2], Trisha Seal [3], Samaya Danthurthy [4], Julia Zaias [5], Keiran S. M. Smalley [6] and Dmitriy Minond [1,2,*]

1 Rumbaugh-Goodwin Institute for Cancer Research, Nova Southeastern University, 3321 College Avenue, CCR r.605, Fort Lauderdale, FL 33314, USA
2 Department of Pharmaceutical Sciences, College of Pharmacy, Nova Southeastern University, 3321 College Avenue, Fort Lauderdale, FL 33314, USA
3 Halmos College of Arts and Sciences, Nova Southeastern University, 3301 College Avenue, Fort Lauderdale, FL 33314, USA
4 Honors College, Nova Southeastern University, 8000 N Ocean Dr., Dania Beach, FL 33004, USA
5 Division of Comparative Pathology, University of Miami, 1501 NW 10th Ave, Miami, FL 33136, USA
6 Moffitt Cancer Center, Department of Tumor Biology, 12902 Magnolia Drive, Tampa, FL 33612, USA
* Correspondence: dminond@nova.edu

Supporting Information

Results of NMR and LCMS characterization of lead compounds.

14

(S)-1((R)-1((S)-2-(((S)-6-benzyl-2,3-dioxopiperazin-1-yl)methyl)pyrrolidin-1-yl)-3-cyclohexylpropan-2-yl)-5-(4-hydroxybenzyl)-4-phenethylpiperazine-2,3-dione (14). Using General Scheme (scheme 1) for the synthesis of compound 14 was synthesized using the following reagents: Boc-L-Phenylalanine -OH (R1), Boc-D-Cyclohexylalanine-OH (R2), Boc-L-Tyrosine(2-Br-Z)-OH (R3), and Phenylacetic Acid (R4). The final crude product was purified using HPLC as described above, with a gradient of (B) 0/5, 2/5, 4/20, 40/55. 1H NMR (400 MHz, DMSO-d6) δ ppm 0.80 - 1.05 (m, 2 H) 1.08 - 1.20 (m, 3 H) 1.24 (br. s., 2 H) 1.50 - 1.71 (m, 6 H) 1.78 - 1.96 (m, 2 H) 1.97 - 2.13 (m, 2 H) 2.55 - 2.65 (m, 2 H) 2.67 - 2.88 (m, 6 H) 2.90 - 3.04 (m, 3 H) 3.11 (d, J=12.96 Hz, 1 H) 3.19 - 3.32 (m, 2 H) 3.37 (br. s., 1 H) 3.52 (dd, J=13.08, 3.30 Hz, 2 H) 3.65 (d, J=10.03 Hz, 2 H) 3.70 - 3.94 (m, 2 H) 6.73 (m, J=8.07 Hz, 2 H) 7.01 (m, J=8.07 Hz, 2 H) 7.15 - 7.31 (m, 9 H) 8.50 (d, J=5.01 Hz, 1 H) 13C NMR (101 MHz, DMSO-d6) δ ppm 22.74 (s, 1 C) 26.13 (s, 1 C) 26.30 (s, 1 C) 26.56 (s, 1 C) 29.54 (s, 1 C) 32.83 (s, 1 C) 33.55 (s, 1 C) 33.63 (s, 1 C) 34.51 (s, 1 C) 37.14 (s, 1 C) 37.51 (s, 1 C) 39.40 (s, 1 C) 39.61 (s, 1 C) 39.82 (s, 1 C) 40.03 (s, 1 C) 40.23 (s, 1 C) 40.45 (s, 1 C) 40.66 (s, 1 C) 48.41 (s, 1 C) 55.86 (s, 1 C) 58.51 (s, 1 C) 62.76 (s, 1 C) 115.89 (s, 1 C) 126.79 (s, 1 C) 127.11 (s, 1 C) 127.76 (s, 1 C) 128.81 (s, 1 C) 128.98 (s, 1 C) 129.13 (s, 1 C) 129.77 (s, 1 C) 130.35 (s, 1 C) 137.72 (s, 1 C) 139.29 (s, 1 C) 156.65 (s, 1 C) 156.72 (s, 1 C) 157.34 (s, 1 C) 157.80 (s, 1 C) 157.95 (s, 1 C) m/z calcd C44H55N5O5 [M+H]+ 734.4281, found 735.1890 (MALDI), 734.15 (MS) Purity LCMS: 98.0% (TIC), 99.0% (254 nm, peak height).

18

(S)-4-(2-(adamantan-1yl)ethyl)-1-((S)-1-((S)-2-(((S)-6-benzyl-2,3-dioxopiperazin-1-yl)methyl)pyrrolidin-1-yl)-3-phenylpropan-2-yl)-5-(4-hydroxybenzyl)piperazine-2,3-dione (18). Using General Scheme (scheme 1) for the synthesis of compound 18 was synthesized using the following reagents: Boc-L-Phenylalanine -OH (R1), Boc-L-Phenylalanine -OH (R2), Boc-L-Tyrosine(2-Br-Z)-OH (R3), and 1-Adamantaneacetic Acid (R4). The final crude product was purified using HPLC as described above, with a gradient of (B) 0/5, 2/5, 4/30, 40/65. 1H NMR (400

MHz, DMSO-d6) δ ppm 1.06 - 1.32 (m, 2 H) 1.38 (br. s., 5 H) 1.48 - 1.60 (m, 3 H) 1.61 - 1.73 (m, 4 H) 1.74 - 1.86 (m, 1 H) 1.86 - 2.02 (m, 3 H) 2.25 (q, J=8.15 Hz, 1 H) 2.38 (d, J=7.46 Hz, 2 H) 2.61 (br. s., 2 H) 2.75 - 3.02 (m, 6 H) 3.12 (br. s., 1 H) 3.22 (d, J=12.10 Hz, 2 H) 3.29 - 3.42 (m, 7 H) 3.42 - 3.63 (m, 4 H) 3.66 - 3.83 (m, 2 H) 3.88 (d, J=10.51 Hz, 1 H) 6.76 (d, J=8.07 Hz, 2 H) 7.02 (d, J=6.97 Hz, 3 H) 7.09 - 7.34 (m, 6 H) 8.54 (d, J=5.13 Hz, 1 H) 13C NMR (101 MHz, DMSO-d6) δ ppm 23.36 (s, 1 C) 28.37 (s, 1 C) 31.86 (s, 1 C) 36.89 (s, 1 C) 36.98 (s, 1 C) 39.40 (s, 1 C) 39.61 (s, 1 C) 39.82 (s, 1 C) 40.03 (s, 1 C) 40.23 (s, 1 C) 40.45 (s, 1 C) 40.66 (s, 1 C) 41.13 (s, 1 C) 41.96 (s, 1 C) 54.85 (s, 1 C) 56.35 (s, 1 C) 58.48 (s, 1 C) 63.97 (s, 1 C) 114.23 (s, 1 C) 115.97 (s, 1 C) 126.74 (s, 1 C) 127.20 (s, 1 C) 127.73 (s, 1 C) 128.72 (s, 1 C) 128.79 (s, 1 C) 129.08 (s, 1 C) 129.78 (s, 1 C) 130.46 (s, 1 C) 137.20 (s, 1 C) 137.91 (s, 1 C) 138.69 (s, 1 C) 144.44 (s, 1 C) 156.40 (s, 1 C) 156.77 (s, 1 C) 157.84 (s, 1 C) 158.36 (s, 1 C) 165.03 (s, 1 C) 174.17 (s, 1 C) 176.50 (s, 1 C) 176.94 (s, 1 C) m/z calcd C48H59N5O5 [M+H]+ 786.4594, found 787.0037 (MALDI), 786.15 (MS). Purity LCMS: 97.1% (TIC), 98.0% (254nm, peak height).

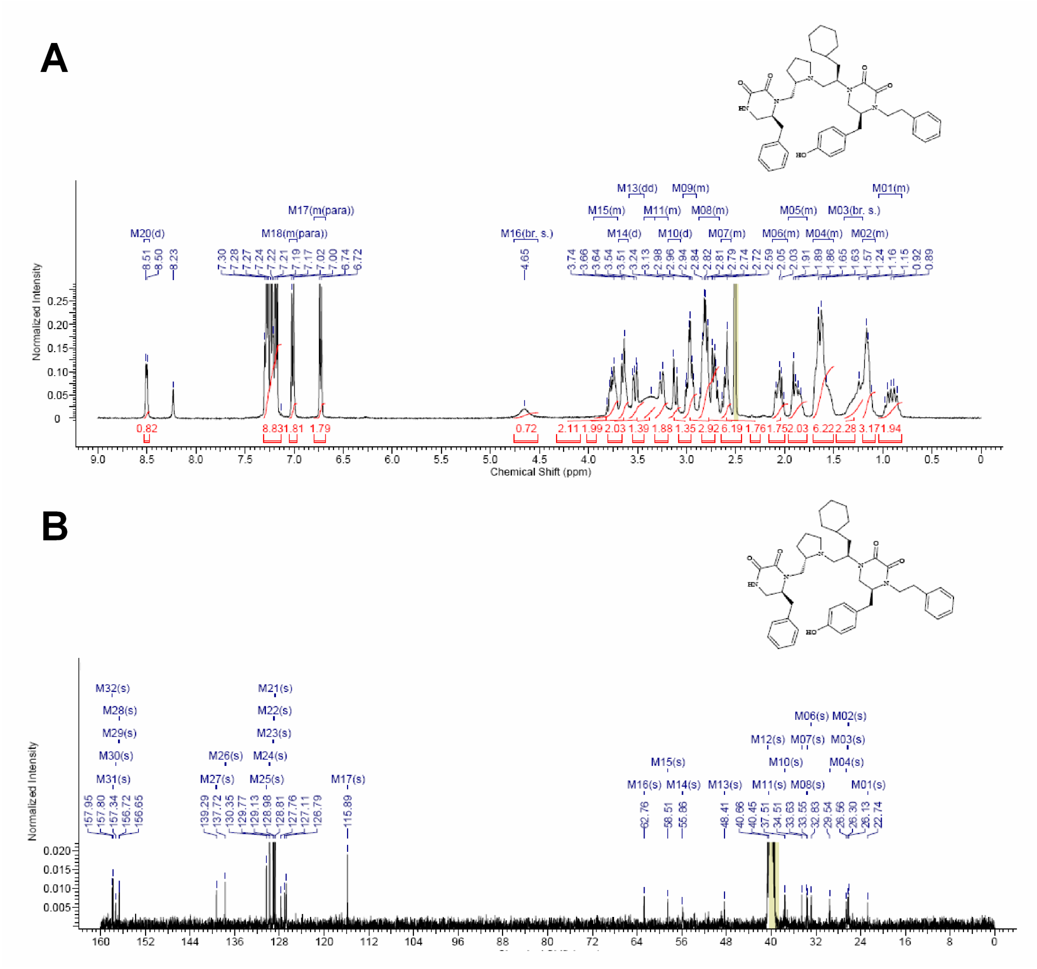

Supporting Information Figure S1. NMR characterization of compound 14. (**A**) 1H NMR spectra of compound 14; (**B**) 13C NMR spectra of compound 14.

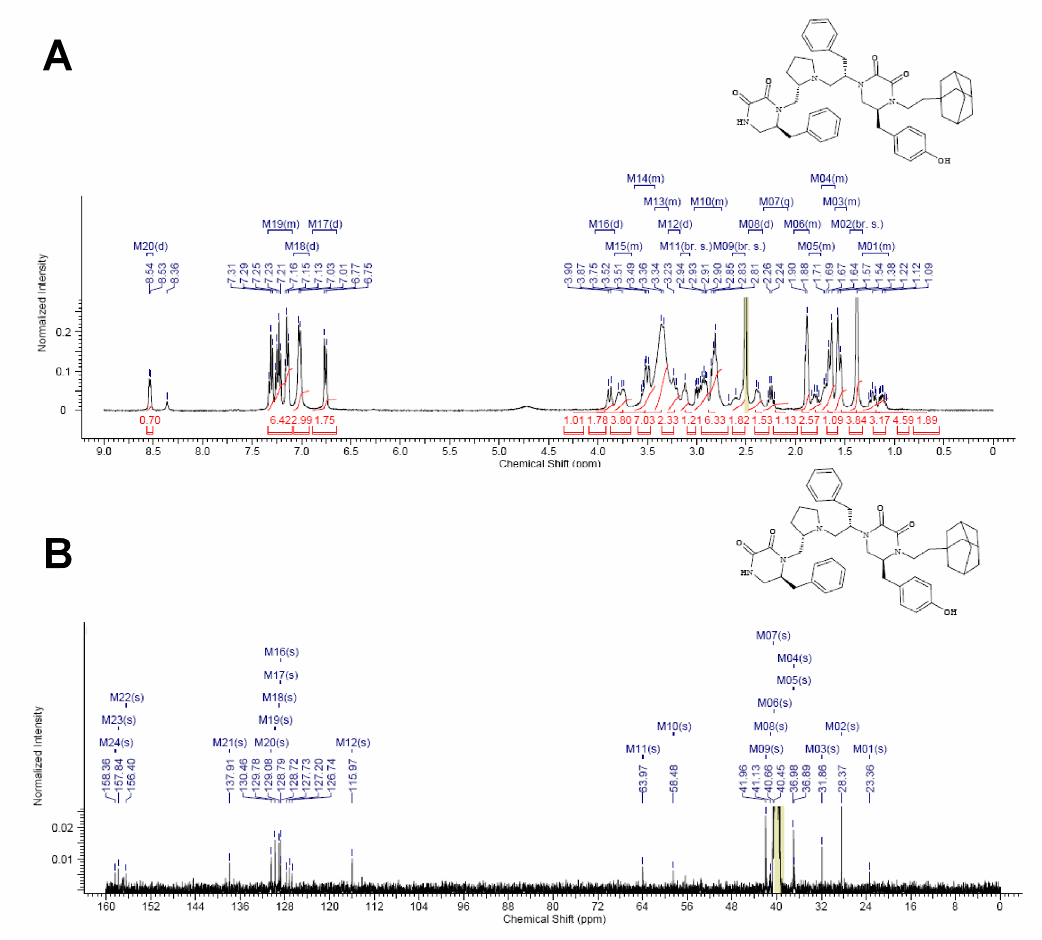

Supporting Information Figure S2. NMR characterization of compound 18. (**A**) 1H NMR spectra of compound 18; (**B**) 13C NMR spectra of compound 18.
